# Reduction of deep surgical site infections in cardiac surgery by introducing a multimodal infection control program

**DOI:** 10.1186/cc14159

**Published:** 2015-03-16

**Authors:** A Rutten, JP Ory, L Jamaer, A Van Assche, J Dubois

**Affiliations:** 1 Jessa Ziekenhuis, Hasselt, Belgium

## Introduction

Deep surgical site infections (DSSI) are a major complication after cardiac surgery with a high mortality rate and reported incidences between 0.5 and 5%. Implementing a comprehensive infection control program (ICP) reduces this incidence [1]. The incidence in our hospital varied from 3.1 to 3.8%, which was considered too high. We evaluated the impact of introducing a multimodal ICP on the incidence of DSSI.

## Methods

**Figure 1:**
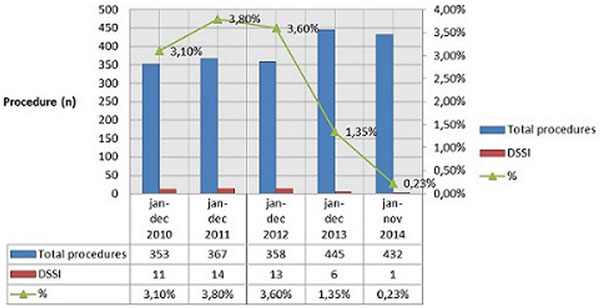
**Incidence of DSSI**.

We noticed a too high incidence of DSSI after cardiac surgery during an observational 3-year period (Figure 1). In February 2013 we introduced a bundle of interdisciplinary infection control measures. Medical and nursing staff of all involved departments took part in developing and implementing these guidelines. Besides emphasizing the importance of existing guidelines (antiseptic shower, hair removal by clipper, strict hand hygiene, prophylactic antibiotics, limiting OR traffic, tight glycemic control (80 to 110 mg/ dl), and so on), new strategies were introduced. The most important new strategies were nasal decolonization with mupirocin twice daily 48 hours perioperatively, preoperative antiseptic skin preparation twice (chlorhexidine gluconate 0.5%), applying topical skin adhesive to the sternal wound postoperatively and in the case of CABG procedures maintaining a strict barrier between the vein harvesting procedure and the chest procedure.

## Results

We observed a significant reduction in DSSI rates in cardiac surgery following implementation of a multimodal ICP from 3.1% in 2010 down to 0.23% in November 2014 (Figure 1).

## Conclusion

Implementing a multimodal ICP significantly reduced the incidence of DSSI in our hospital but it remains difficult to identify which interventions were most effective.

## References

[B1] Guide for the prevention of mediastinitis surgical site infections following cardiac surgery 2008 http://www.apic.org

